# P-1418. Risk for Early and Late Readmission for Serious Injection-Related Infections: the CHOICE+ Cohort

**DOI:** 10.1093/ofid/ofae631.1593

**Published:** 2025-01-29

**Authors:** Edward C Traver, Habib Omari, Jasmine Stevens, Meghan Derenoncourt, Hannah E Flores, Ishan Kumar Vaish, Sumitha Raman, Ayako Wendy Fujita, Becky Reece, Irene Kuo, Alaina Steck, Jillian S Catalanotti, Joseph E Carpenter, Sarah Kattakuzhy, Elana S Rosenthal

**Affiliations:** University of Maryland School of Medicine, Baltimore, MD; University of Maryland Baltimore, Baltimore, Maryland; University of Maryland School of Medicine, Baltimore, MD; University of Maryland, Baltimore, Baltimore, Maryland; University of Maryland, Baltimore, Maryland; University of Maryland Medical School, Baltimore, Maryland; George Washington University, Washington, District of Columbia; Emory University School of Medicine, Atlanta, Georgia; West Virginia University, Morgantown, WV; George Washington University Milken Institute School of Public Health, Washington, District of Columbia; Emory University, Atlanta, Georgia; The George Washington University of Medicine and Health Sciences, Washington, District of Columbia; Emory University School of Medicine, Atlanta, Georgia; Institute for Human Virology (IHV), University of Maryland School of Medicine, Baltimore, Maryland; Institute for Human Virology (IHV), University of Maryland School of Medicine, Baltimore, Maryland

## Abstract

**Background:**

People who inject drugs hospitalized with severe injection-related infections (SIRI) face barriers to antibiotic treatment completion and remain at risk for new infections due to ongoing injection drug use. We explored risk factors for SIRI readmission and whether readmissions were caused by the index SIRI or a new SIRI.

**Figure:**
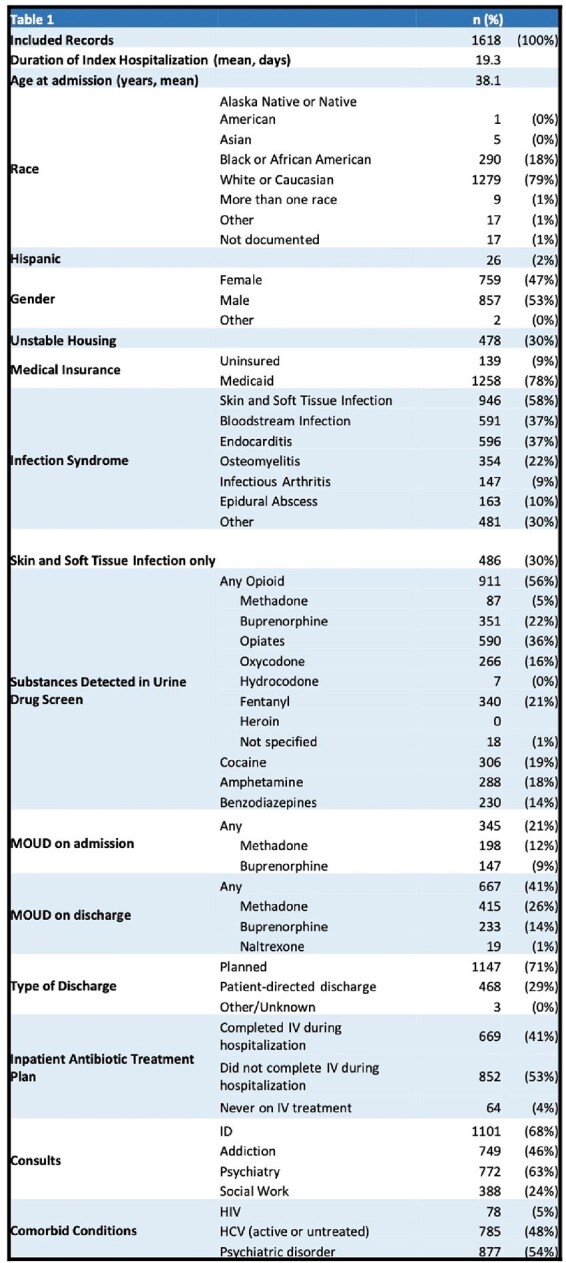

**Methods:**

CHOICE+ is a retrospective cohort study of adults hospitalized with SIRI due to injection opioid use at 4 hospital systems between 1/1/2018 and 3/31/2022. Data were collected by abstraction of the medical record. Primary outcomes were readmission for SIRI within 1 year and time to readmission. Categorical variables were analyzed by chi-square, Fischer exact test, and multivariate logistic regression. Time to readmission was compared by the Mann-Whitney test.

**Figure:**
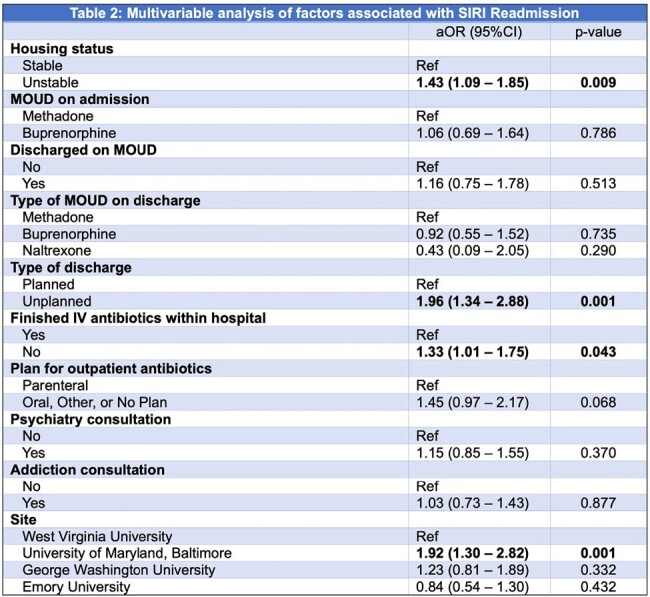

**Results:**

Of 1618 patients discharged alive, 439 (27%) had ≥1 readmission for SIRI. Unstable housing, patient-directed discharge, and not finishing planned intravenous antibiotics in the hospital were associated with increased risk for SIRI readmission (Table 2). Risk of SIRI readmission was not associated with addiction or psychiatry consultation, discharge on medication for opioid use disorder (MOUD), or type of MOUD. The cause of readmission in 238 (54%) was the index SIRI and in 201 (46%) was a new SIRI. Median time to readmission was shorter for those readmitted with index SIRI compared to a new SIRI (10 v. 126 days, p < 0.0001, Figure 1) and for those who did not complete treatment for the index SIRI compared to those who completed treatment (7 v. 78 days, p < 0.0001, Figure 2).

**Figure:**
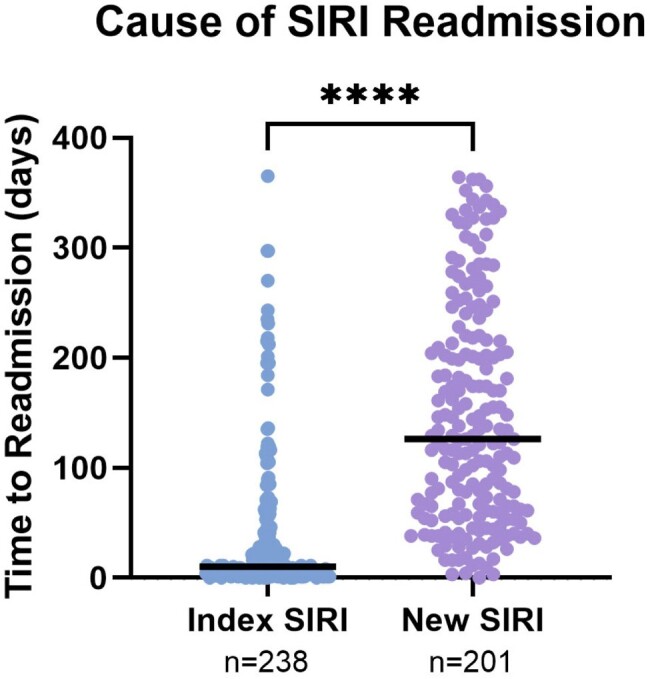

**Conclusion:**

In this large, representative study, readmission for SIRI occurred frequently for both problems related to the index SIRI as well as development of new SIRI. Readmission for index SIRI occurred earlier than for new SIRI, especially for those who did not complete antibiotic treatment. Notably, readmission for SIRI was not associated with inpatient interventions to address addiction during the index hospitalization. Therefore, prioritizing completion of treatment of the index SIRI may be critical for reducing early readmissions. In contrast, prevention of new SIRI, by longitudinally addressing addiction, social determinants of health, and harm reduction, may be necessary to reduce later readmissions.

**Figure:**
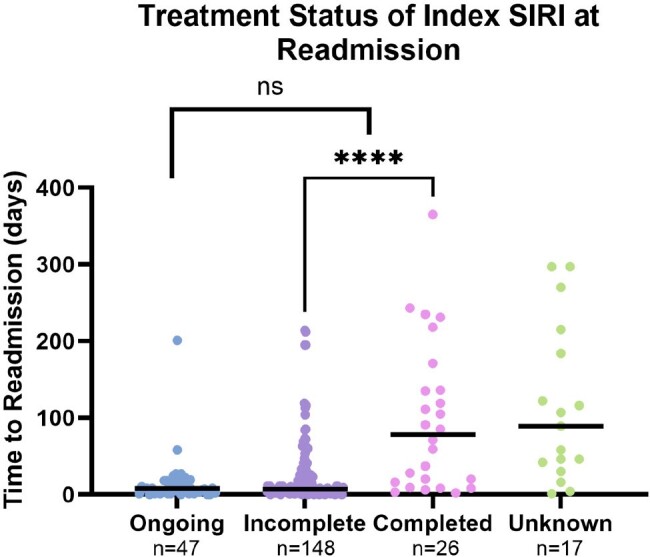

**Disclosures:**

**Elana S. Rosenthal, MD**, Gilead Sciences: Grant/Research Support|Merck: Grant/Research Support

